# Local application of bacteria improves safety of *Salmonella*-mediated tumor therapy and retains advantages of systemic infection

**DOI:** 10.18632/oncotarget.18392

**Published:** 2017-06-07

**Authors:** Dino Kocijancic, Sebastian Felgner, Tim Schauer, Michael Frahm, Ulrike Heise, Kurt Zimmermann, Marc Erhardt, Siegfried Weiss

**Affiliations:** ^1^ Molecular Immunology, Helmholtz Center for Infection Research, Braunschweig, Germany; ^2^ Infection Biology of Salmonella, Helmholtz Center for Infection Research, Braunschweig, Germany; ^3^ Mouse-Pathology Service Unit, Helmholtz Center for Infection Research, Braunschweig, Germany; ^4^ Symbio Gruppe GmbH & Co KG, Herborn, Germany; ^5^ Institute of Immunology, Medical School Hannover, Hannover, Germany

**Keywords:** Intra-tumoral injection, Salmonella, E. coli, bacteria mediated tumor therapy, murine tumor model

## Abstract

Cancer is a devastating disease and a large socio-economic burden. Novel therapeutic solutions are on the rise, although a cure remains elusive. Application of microorganisms represents an ancient therapeutic strategy, lately revoked and refined via simultaneous attenuation and amelioration of pathogenic properties. *Salmonella* Typhimurium has prevailed in preclinical development. Yet, using virulent strains for systemic treatment might cause severe side effects. In the present study, we highlight a modified strain based on *Salmonella* Typhimurium UK-1 expressing hexa-acylated Lipid A. We corroborate improved anti-tumor properties of this strain and investigate to which extent an intra-tumoral (i.t.) route of infection could help improve safety and retain advantages of systemic intravenous (i.v.) application. Our results show that i.t. infection exhibits therapeutic efficacy against CT26 and F1.A11 tumors similar to a systemic route of inoculation. Moreover, i.t. application allows extensive dose titration without compromising tumor colonization. Adverse colonization of healthy organs was generally reduced via i.t. infection and accompanied by less body weight loss of the murine host. Despite local application, adjuvanticity remained, and a CT26-specific CD8^+^ T cell response was effectively stimulated. Most interestingly, also secondary tumors could be targeted with this strategy, thereby extending the unique tumor targeting ability of *Salmonella*. The i.t. route of inoculation may reap the benefits of systemic infection and aid in safety assurance while directing potency of an oncolytic vector to where it is most needed, namely the primary tumor.

## INTRODUCTION

Cancer ranks among the diseases that have experienced the least form of improvements in prevention and therapy over the last century based on incidence- and mortality rates [1]. Therefore it retains a position as the second most frequent cause of death, with no cure available to date [2]. This threat is imminent due to an increasing incidence with age, and an expanding elderly population [3, 4]. In addition, cancer represents a great socio-economic burden [5]. For these reasons, novel solutions like immune therapies are under thorough development. However, a cost-efficient, effective and general type of cancer therapy has yet to advance through the drug development chain and replace conventional solutions.

The intentional use of infectious agents in biomedicine may be perceived as a first generation type of cancer immune therapy. William B. Coley, as one of the pioneers, propagated this type of therapy in the early 20^th^ century [6–8]. Although he gained impressive results, his findings were undermined by skepticism among opinion leaders and the forthcoming alternative radiotherapy [9, 10]. Therefore, this therapeutic option received inferior attention throughout the last century. Inability to control side effects of infection and to provide a reasonable explanation for the mode of action were major concerns for this therapy at that time.

Since the dawn of molecular genetics, safety shortcomings have been addressed by means of bacterial attenuation [11–13]. However, the problematic inverse connection between safety and therapeutic potency remains a hurdle in strategies deployed [10]. Thus, many efforts focus on tailoring the intrinsic therapeutic effect via alteration of immunogenic components like the lipopolysaccharide (LPS) of Gram-negative bacteria [13–17]. However, this intrinsic therapeutic potential may be limited and might not be reconcilable with safety. Therefore, new options of safety assurance need to be explored.

In recent time, the predominant focus in bacteria mediated tumor therapy (BMTT) has been placed on the Gram-negative bacterium *Salmonella enterica* serovar Typhimurium (*S*. Typhimurium) [18]. The advantage of *Salmonella* includes an intrinsic therapeutic effect and a unique ability to specifically colonize tumors [19–22]. The latter has also been exploited as strategy to deliver genetically encoded cargo directly into the tumor [23–31]. Numerous innovative examples and different designs highlight the versatile potential of such bacterial vectors as a highly promising cancer-therapeutic solution [32, 33].

The trend in BMTT has been to administer *S*. Typhimurium by intravenous infection. Numerous groups have consistently shown in a wide range of preclinical models that *Salmonella* is able to colonize cancerous tissue specifically with ratios of more than 1000:1 compared to healthy tissues such as liver and spleen [34–37]. However, bacteria of above 10^6^ CFU per gram tissue in healthy organs may restrict dosing regimens and inflict severe side effects. On the other hand, avoiding such problems by excessive attenuation of *Salmonella* has been proven to cause loss of intrinsic potency *in vivo* as demonstrated in clinical trials with *Salmonella* VNP20009 [38].

The unique abilities of *Salmonella* are important benefits for BMTT. However, whether the ability to target tumors is inevitably tied to a systemic mode of application remains questionable. William Coley applied his bacterial therapy locally with great efficiency, albeit for reasons of practicality and safety [7, 9]. While researchers nowadays seek to ensure safety via genetic manipulation, local applications may still provide a suitable strategy to retain virulence while exhibiting a better safety profile. However, important criteria for successful local application should include: i) preserved advantages of systemic infection, ii) improved efficacy per comparable dose, and iii) a better safety profile.

We have extensively characterized therapeutic mechanisms of several variants of *S*. Typhimurium upon systemic infection [21, 37, 39–41]. In the present study, we use the same murine tumor infection model to perform a direct comparison between a systemic intravenous (i.v.) and local intra-tumoral (i.t.) route of infection with our most current attenuated *Salmonella* variants. In particular, we focus on i) safety aspects, including the ability to limit dissemination, and ii) effectiveness in propagating therapeutic effects in primary and secondary tumors, when administered locally. Overall, our study demonstrates that an i.t. route of infection indeed can be used to increase the safety of BMTT by minimizing dissemination and reducing inoculum size without compromising the overall therapeutic effectiveness.

## RESULTS

### Local infection retains a tumor therapeutic effect

The therapeutic potential of our hexaacylated Lipid A mutant ‘SF100’ harboring mutations *ΔlpxR9, ΔpagL7 and ΔpagP8* and the influence of *ΔaroA* to this *Salmonella* vector have been thoroughly described in our recent publications [14, 41]. In the present study, we corroborated a new strain 'sF200‘, built on the above modifications and carrying additional mutations *ΔydiV* and *ΔfliF*. These genes encode a negative regulator of flagella synthesis [42, 43] and a membrane bound protein required for flagellar synthesis [44, 45], respectively.

**Figure 1:**
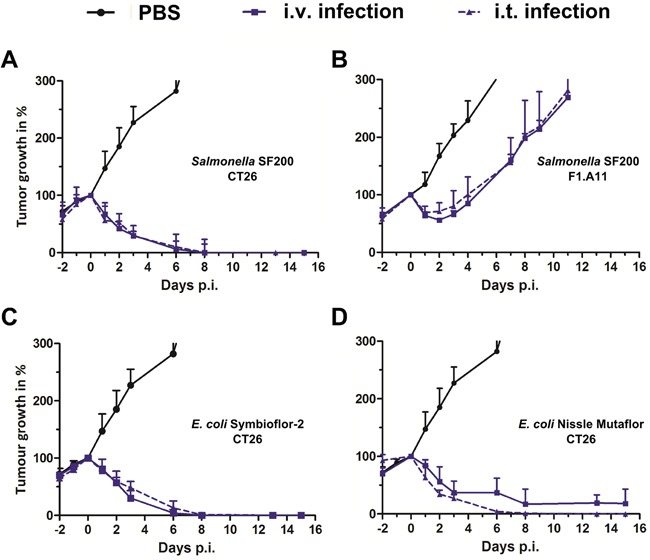
Tumor development upon intravenous and intra-tumoral infection with *Salmonella* and probiotic *E. coli* CT26 tumor-bearing mice were infected with 5×10^6^ CFU SF200 (*ΔlpxR9 ΔpagL7 ΔpagP8 ΔaroA ΔydiV ΔfliF*) **(A)**, Symbioflor-2 **(C)** and *E. coli* Nissle **(D)**. Considering more resilient tumors, F1.A11 tumor-bearing mice were infected with 5×10^6^ SF200 **(B)**. Straight lines depict i.v. infection and dotted lines i.t. infection. Tumor volumes were calculated on the basis of caliper measurements. PBS served as a negative control. Displayed are values of mean ± SEM. Results are representative of two independent experiments with five replicates in each group.

To evaluate the performance of this therapeutic strain *in vivo*, an inoculum of 5×10^6^ bacteria was administered to syngeneic tumor bearing BALB/c mice by intravenous (i.v.) or intra-tumoral (i.t.) injection. The same infection dose was applied in most of our previous studies, and thus allows direct comparison [13, 37, 40, 46, 47]. Tumor development was assessed over a period of two weeks, or until the reach of a humane endpoint. As seen in Figure 1A, the *Salmonella* variant SF200 (*ΔlpxR9 ΔpagL7 ΔpagP8 ΔaroA ΔydiV ΔfliF*) induced complete clearance of CT26 tumors by 15 days of infection. The kinetic of tumor regression was comparable between both routes of inoculation (Figure 1A). To corroborate results, we repeated our experiment with the more resistant fibro-sarcoma cell line F1.A11 [13]. Here, SF200 induced initial retardation, however tumors started to outgrow after day 3 post infection (Figure 1B). Interestingly, this profile was observed regardless the route of infection, and may suggest that systemic infection is not essential for induction of a therapeutic effect.

To generalize this effect, we compared the therapeutic potency of probiotic *E. coli* upon i.t. and i.v. infection. The *E. coli* probiotics Symbioflor-2 (G1 - G10) and Mutaflor (*E. coli* Nissle, EcN) have been explored for tumor therapy on several occasions and shown to exhibit inferior intrinsic potency in the CT26 model system compared to *Salmonella* Typhimurium [21, 47]. Here, tumor development displayed a similar profile upon Symbioflor-2 infection between i.t. and i.v. inoculation (Figure 1C). The efficacy of EcN was even mildly improved upon i.t. infection, causing faster regression and complete clearance in the experimental group (Figure 1D).

**Figure 2:**
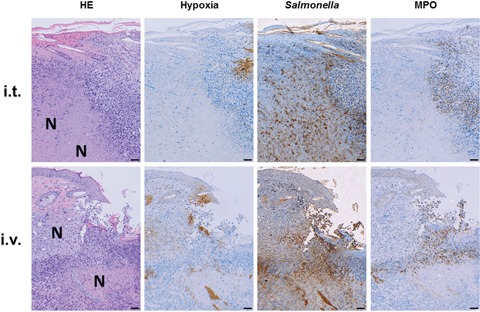
Intra-tumoral inoculation of *Salmonella* causes manifestations in the CT26 tumor similar to systemic infection CT26 tumor-bearing mice were infected with 5×10^6^ CFU SF200 (*ΔlpxR9 ΔpagL7 ΔpagP8 ΔaroA ΔydiV ΔfliF*) via i.t. and i.v. routes of inoculation (top and bottom row, resp.). 48 hpi, tumors were isolated, embedded in paraffin and prepared for immune histochemical staining. Similar histological profiles between i.t. and i.v. infections: similar degree of necrosis formation and hypoxia, dispersion of salmonellae in and beyond necrotic center, and presence of neutrophils in immediate proximity to the salmonellae. Images displayed are representative of four replicates. “N” denotes areas of necrosis. Hypoxia was stained with antibodies against metabolites of pimonidazole-HCl administered i.v. 30 mins prior to isolation. Myeloperoxidase (MPO) denotes presence of neutrophilic granulocytes, and salmonellae was stained using a specific antibody. Differential staining was performed on consecutive sections. Scale bar corresponds to 100 μm. Images representative of at least 3 replicates are displayed.

Immune histology was performed to extend our comparison. As expected, the microscopic profile remained similar between both routes of infection. All tumors developed necrosis and hypoxic regions by 24 hours post infection (hpi) (Supplementary Figure 1), which was even more prominent after 48 hpi (Figure 2). This niche was occupied by salmonellae, and surrounded by granulocytic neutrophils. The phenotypic characteristics were similar to earlier reports involving *Salmonella* variants [40, 48], and herewith validated with our most recent strain SF200, and the intra-tumoral route of infection.

In summary, direct bacterial inoculation into the target tumor retains intrinsic therapeutic potency as a customary intravenous route of infection.

### Reduced dissemination and improved health status

**Figure 3:**
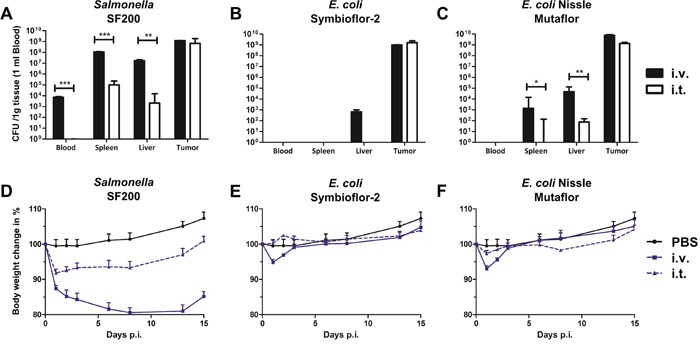
Safety evaluation upon intravenous and intra-tumoral infection with *Salmonella* and probiotic *E. coli* CT26 tumor bearing mice were infected i.v. and i.t. with 5×10^6^ CFU SF200 (*ΔlpxR9 ΔpagL7 ΔpagP8 ΔaroA ΔydiV ΔfliF*) and probiotic *E. coli*. **(A-C)** Blood, spleen, liver and tumor were analyzed for bacterial burden by plating serial dilutions of tissue homogenates. CFU counts were determined 48 hpi. Significantly lower numbers were observed during i.t. infections. **(D-F)** Body weight as indicator for the general health status of mice. Again, i.t. infection resulted in reduced body weight loss. PBS served as negative control. Displayed are medians with range. Results are representative of two independent experiments with five replicates per group. *, p<0.05; **, p<0.01; ***, p<0.001.

Tumor colonization remains a favorable feature of *Salmonella* because of direct oncolytic effects, either intrinsically preserved or reinforced through delivery of genetic cargo [31, 49–51]. Therefore, we tested our *Salmonella* strain SF200 for its intrinsic ability of tumor targeting upon systemic infection, as it had previously been reported for its parental strain [14, 52]. We also compared it to colonization profiles upon i.t. infection. The i.t. route of bacterial inoculation should minimize dissemination and thus cause a safer phenotype for the host. As expected, SF200 did colonize the tumor with above 1×10^8^ CFU/g tumor by 36 hpi upon systemic infection (Figure 3A). I.t. application resulted in similar high CFU in the tumor. As hypothesized, local inoculation did minimize dissemination. CFU in spleen and liver were reduced by a factor of 1×10^3^ and 1×10^4^, respectively, compared to i.v. infection. At this time point, lack of CFU in blood confirmed the absence of circulating salmonellae. The safer colonization profile was also reflected in the body weight loss, i.e. reflecting the general health status, of the hosts. I.t. Infection caused a milder initial drop followed by quick recovery after 1 dpi (Figure 3D). These results were corroborated with the probiotic strains of *E. coli*. Although a significant reduction in adverse colonization was evident with both Symbioflor-2 and EcN upon i.t. infection (Figure 3B, 3C), the impact on host body weight was less prominent compared to *Salmonella* (Figure 3E, 3F). The latter could be explained by a general superior health status upon systemic infection with probiotic bacterial strains [22, 47].

Overall, an intra-tumoral route of inoculation does restrict dissemination of *Salmonella*, and thus improves the overall health status of murine subjects during BMTT.

### Safety and early tumor colonization with inoculum dose variations

For downstream clinical trials, dose escalation to install effects could become an important differentiator. This was exemplified in the former clinical trial using VNP20009, where toxicity prohibited higher dosing, and thereby also potential therapeutic effects [38]. In addition, we speculate that reduced side effects via an i.t. route of infection may accommodate the use of more virulent, and thus therapeutically more potent, *Salmonella* variants. Nevertheless, placing emphasis on the influence of route of infection, we allowed the use of a more attenuated *Salmonella* variant SF201 (*ΔlpxR9 ΔpagL7 ΔpagP8 ΔaroA ΔssrA*) for dose titration. This strain contains the same Lipid A structure as the *Salmonella* strain SF200, although phenotypically less virulent by the deletion of the *Salmonella* Pathogenicity Island-2 (SPI-2) gene *ssrA* [53]. To investigate the colonization over longer time we applied the F1.A11 tumor model which is known to be less prone to clearance [13].

**Figure 4:**
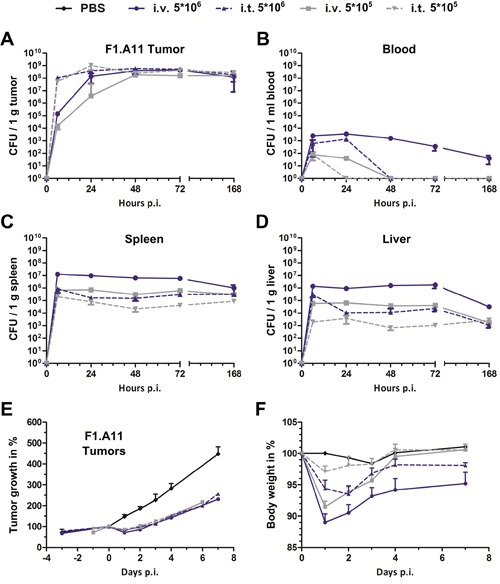
Intra-tumoral infection with *Salmonella* promotes higher tumor specificity and faster tumor colonization even at lower doses CT26- tumor bearing mice were infected i.v. and i.t. with a dose of 5×10^6^ and 5×10^5^ *Salmonella* variant SF201 (*ΔlpxR9 ΔpagL7 ΔpagP8 ΔaroA ΔssrA:*:Km). Tumors **(A)**, blood **(B)**, spleen **(C)** and liver **(D)**. Bacterial burden was determined by plating serial dilutions of tissue homogenates. CFU were analyzed 36 hpi. **(E)** Tumor volumes were calculated on the basis of caliper measurements following infection with SF201. PBS served as negative control. **(F)** Body weight measurement served as indicator for the general health status. I.t. infection resulted in a significantly reduced bacterial burden and body weight decrease. PBS served as negative control. Displayed are medians with range. Results are representative of two independent experiments with five replicates per group.

Overall, a 10-fold reduction of the inoculum (i.e. 5×10^5^ CFU) significantly reduced the side effects during i.t infection while preserving therapeutic potency (Figure 4). In comparison to i.v. infection, i.t. application allowed for 10-fold higher dosing, without surpassing CFU counts in blood, spleen and liver (Figure 4B–4D). A similar tendency was reflected in the host body weight, which was significantly less affected by dosing via an i.t. route of infection (Figure 4F). Interestingly, further down titration to an infectious dose of 5×10^3^ allowed significant bacterial clearance from healthy organs without compromising maximum CFU in the tumor (Supplementary Figure 2). Conversely, intravenous infection caused significant delay in tumor colonization with already a 10-fold reduced i.v. inoculum. Moreover, tumor colonization was completely abrogated when a 1000-fold lower dose of 5×10^3^ was applied (Figure 4A, Supplementary Figure 2).

Of note, the localized intra-tumoral route of infection displayed greater plasticity for tumor colonization with regards to size of inoculum. Consequently, dose reduction facilitated a greater level of safety. Considering therapeutic strategies based on delivery of therapeutic cargo, this route of inoculation may allow altered dosing regimens, and thus prime BMTT for success in clinical trials.

### Bacterial application i.t. induces an effective adaptive anti-tumor immune response against CT26

We have previously reported that i.v. infection with strains of *Salmonella* can induce a memory immune response against CT26. The effector mechanism mainly involves CD8^+^ T cells [21, 47]. Therapeutic potency by i.t. infection as seen in Figure 1 implies that such an adjuvant effect may be preserved.

**Figure 5:**
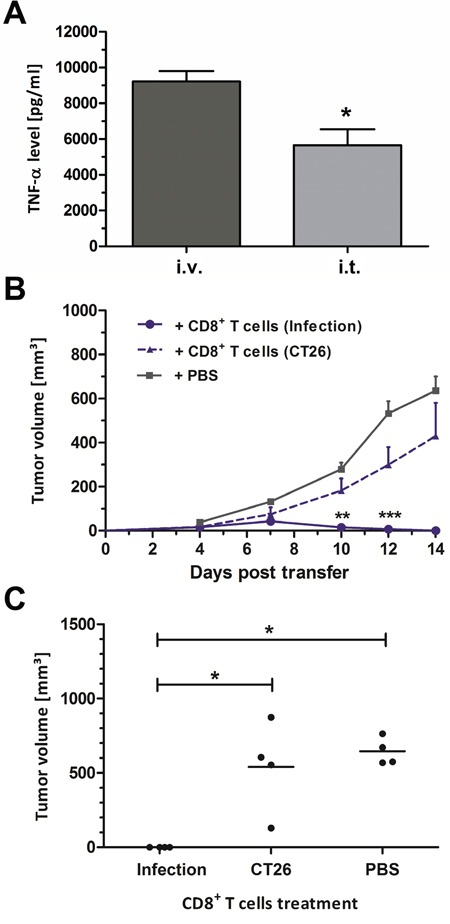
Intra-tumoral infection induces innate and adaptive immune responses **(A)** TNF-α levels in sera of CT26 tumor bearing mice isolated 1.5 h after infection with SF200 (*ΔlpxR9 ΔpagL7 ΔpagP8 ΔaroA ΔydiV ΔfliF*). **(B)** CT26 tumor development in Rag1^−/−^ mice reconstituted with CD8^+^ T cells at the time of CT26 inoculation. 3×10^6^ CD8^+^ T cells were adoptively transferred from uninfected CT26 bearing mice (“CD8^+^ T cells (CT26)”) or CT26 tumor bearing mice treated with SF200 ((“CD8^+^ T cells (Infection)”). PBS served as negative control. **(C)** Endpoint tumor volume at day 14 post transfer. Displayed are values of mean ± SEM. Results are representative of two independent experiments with five replicates per group. *, p<0.05; **, p<0.01; ***, p<0.001.

TNF-α represents an important readout for the tumor therapeutic effects and a systemic response [35, 54, 55]. Hence, this cytokine was measured in serum upon infection with *Salmonella*. Even though locally administered, i.t. infection induced a strong systemic response of TNF-α at 1.5 hpi (Figure 5A). Albeit significantly reduced compared to an i.v. route of infection, the serum levels obtained may be sufficient to induce global systemic effects, and thereby explain the therapeutic results shown in Figure 1.

To expand on the therapeutic response and to determine whether current dogma of anti-tumoral immune memory applies to i.t. infection, CT26-cleared mice were re-challenged with the same tumor cell line. These tumors did not establish, thus indicating that a memory response had been invoked (data not shown). In extension, we reconstituted Rag1^−/−^ mice with CD8^+^ T cells isolated from wild-type BALB/c mice that had cleared CT26 via i.t. infection. Minimal tumor growth was observed by day 7 post transfer, upon which retardation and complete clearance occurred (Figure 5B). Endpoint comparison of tumor volumes emphasizes a statistically significant effect across all replicates compared to controls reconstituted with naive T cells (Figure 5C).

Altogether, local intra-tumoral infection is able to raise a systemic cytokine response and an effective anti-tumor CD8^+^ T cell response against CT26 tumors.

### Secondary tumor targeting is not restricted to a systemic intravenous route of infection

The ability of *Salmonella* to intrinsically colonize CT26 tumors has been described in numerous reports [56–60], and may be exploited to deliver therapeutic cargo to secondary and surgically inaccessible tumors. This ability has been vastly explored via intravenous infections. It provides an important argument for such a route of application. We set out to explore whether SF200 applied i.t. could sufficiently escape the tumor site of inoculation to colonize “secondary” tumors located at different sites. Evidence collected thus far includes colonization of adverse organs, and a window of several hours, where bacteria are detected in the blood circulation upon i.t. infection. These are indications that may hypothetically also allow for colonization of other tumor niches.

**Figure 6:**
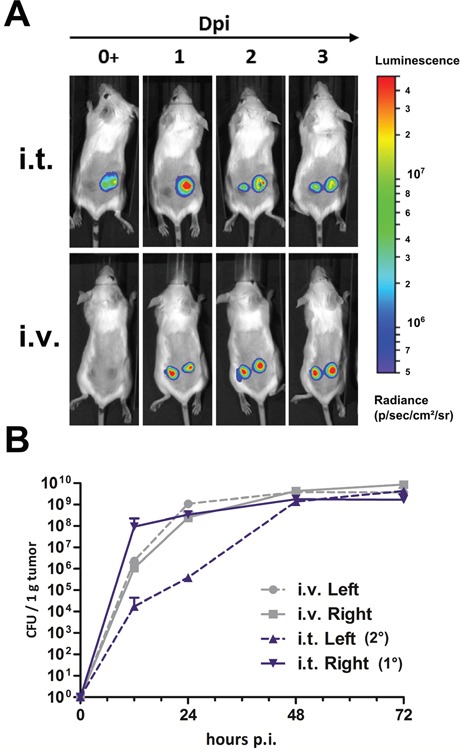
Intra-tumoral infection allows effective colonization of secondary CT26 tumors **(A)** Mice bearing two CT26 tumors were infected i.v. or i.t. into one tumor with 5×10^6^ CFU SF202 (*ΔlpxR9 ΔpagL7 ΔpagP8 ΔaroA ΔydiV ΔfliF* pHL304). Anesthetized mice were analyzed at 0, 1, 2 and 3 dpi using a non-invasive *in vivo* imaging system (IVIS200). **(B)** Bacterial colonization of tumors was determined by plating serial dilutions of tissue homogenates. CFU were analyzed 12, 24, 48 and 72 hpi to match imaging time points. For i.t. infection, primary and secondary tumors are denoted 1° and 2°, respectively. Displayed are medians with range. Results are representative of two independent experiments with five replicates in each group.

To trace the bacteria *in vivo*, we transformed a plasmid encoding the *luxCDABE* operon into our bacteria resulting in SF202 (SF200 + pHL304). This construct ensures constitutive Luciferase (Lux) expression, and is detectable via noninvasive *in vivo* imaging systems [61]. This approach allowed us to track the progression of infection in a subject over time. As expected, Lux signals were detected 1 dpi with equal intensity in two anatomically separated CT26 tumors after i.v. infection (Figure 6A, bottom row). This observation was confirmed by plating (Figure 6B). During i.t. injection, the initial signal in the primary tumors was strong, as confirmed by plating. Interestingly, it was followed by a signal in the anatomically separated tumor at 2 dpi, which further intensified at 3 dpi (Figure 6A, top row). Plating data confirmed the qualitative observation, and revealed bacterial counts of 1×10^4^ CFU per gram in the secondary tumor within 12 hpi (Figure 6B). With the exception of delayed tumor invasion, a plateau of 1×10^8^ CFU per gram in all tumors was substantiated under any circumstance. Similar results were obtained when tumors were placed at a more distant site (i.e. dorsal and abdominal) (data not shown).

Taken together, a superior safety standard, plasticity for dosing, retained adjuvanticity and a preserved ability of targeting secondary tumors, may render intra-tumoral application of bacteria the preferred route of application for progression through clinical trials and treatment of cancer patients with BMTT.

## DISCUSSION

There are several ways to inactivate pathogenic bacteria for clinical application. Heat inactivation and formaldehyde fixation are examples of straightforward techniques, although they might result in reduced efficacy [62, 63]. Thus, bacterial attenuation has become the preferred strategy to accommodate safety. Considering Gram-negative bacterial candidates such as *Salmonella*, they are intrinsically prone to induce septic shock due to their LPS coat [64]. Hence, modifications of LPS represent a rational choice to modify *Salmonella*. In accordance, we have placed extensive emphasis on such modifications to tailor strains of *Salmonella* Typhimurium with balanced properties of safety and intrinsic therapeutic benefit [10, 13]. Modifications shown to improve this balance include a hexa-acylated Lipid A structure implemented by the mutations *ΔlpxR9, ΔpagL7* and *ΔpagP8* [14]. This ensures high affinity binding of Lipid A to TLR4, and thus improved stimulation [65]. Additionally, we could show that the metabolic mutation *ΔaroA* with its long standing tradition provides therapeutic benefit to BMTT [41]. Furthermore, structures like the flagellum, providing an immune stimulatory capacity via TLR5, may contribute relevant therapeutic power [17]. In accordance, our strain SF200 harbored the aforementioned mutations along with a deletion of *ydiV* and *fliF*. In concert, the latter modifications would yield a *Salmonella* strain without functional flagella, however, rich in immune stimulatory flagellar proteins. Overall, this new strain performed well against our murine tumor models (Figure 1). It displayed effectiveness against CT26, and showed even transient therapeutic potency against the more rigid fibrosarcoma cell line F1.A11. The growth of F1.A11 has been reported to be more rigid and unresponsive to *Salmonella* variants like SL7207 [13].

When inoculated intravenously, SF200 still exhibits adverse CFU of above 1×10^6^ in liver and spleen, similar to its parental strain. Therefore, we are convinced that intrinsic attenuation and strain modification cannot per se accommodate therapeutic benefit and safety to a satisfactory level. Nevertheless, possibilities of *Salmonella* as an oncolytic vector that also delivers genetic cargo should be considered as a downstream measure of optimization. Furthermore, strategies of combination therapy [18] or changes in dosing regimens as well as alternate routes of inoculation could aid the optimization process.

Interestingly, a simple strategy to limit dissemination and counteract residual CFU in healthy organs was introduced more than a century ago by William B. Coley. He deployed local bacterial application [6]. This strategy may indeed have been a final resolution for the crude non-modified infectious bacteria at a time where anti-infectives or genetic engineering did not exist. It may now represent a solution to install safety when genetic tailoring has reached its limit. Exploring this strategy by delivering our *Salmonella* strain SF200 locally into the tumor, we were able to limit adverse CFU and install greater safety of treatment without compromising efficacy (Figure 2). When attempting to generalize this method by deploying probiotic *E. coli*, inoculation directly into the tumor, we observed only modestly improved therapeutic effects as seen by clearance of CT26. Hence, we speculate that these bacteria may benefit from a higher initial dose via i.t. infection. This could overload immune escape mechanisms or profit from reduced contact with the innate immune system. Additionally, i.t. injection may cause a wider bacterial spread inside the tumor or simply induce more tissue destruction compared to an i.v. route of delivery [66, 67]. However, the almost negligibly reduced side effects of probiotic vectors upon i.t. infection do not provide an incentive to apply an intra-tumoral route of inoculation with probiotic infection [22, 47, 68]. It could become crucial, however, when sensitivity to human complement would prohibit delivery of the therapeutic vector. As reported in Kocijancic et al. [47], this particular predisposition differentiates probiotic strains of Symbioflor-2 from Mutaflor (*E.coli* Nissle), which were also included in the present study.

The use of *Salmonella* in the clinics may require extensive plasticity in dosing as both safety and benefit needs to be preserved. Our results demonstrate that local intra-tumoral application provides an increased dosing variability compared to i.v. infection. In detail, equally potent tumor colonization over a greater dosing range of 5×10^3^ – 5×10^6^ CFU was achieved with an i.t. application compared to an i.v. route of administration. In addition, side effects i.e. adverse colonization and body weight loss could be minimized via dose titration. We believe that the improved safety profile of i.t. versus i.v. inoculation will more readily allow dose escalation in clinical trials. Altogether, the local intra-tumoral route of application exhibited greater plasticity, however, it needs to be validated with additional close-to-clinic strain candidates and tumor models.

Histological manifestation of BMTT appeared consistent between i.t and i.v. infection. This suggests a similar therapeutic mechanism. The adjuvant effect was inherently preserved with i.t. infection, albeit the systemic cytokine response after i.t. application was slightly inferior to i.v. infection as assessed by levels of serum TNF-α (Figure 5A). Nevertheless, this turned out to be sufficient to install effectiveness against CT26 tumors. Alternatively, i.t. infection may induce significantly higher local levels of such cytokines inside the tumor which could compensate for the systemic deficit. In principle, exaggerated responses induced by i.v. infection might not be required for successful CT26 therapy. Supposedly, even the lower adjuvanticity conferred by intra-tumoral infection or probiotic infection is sufficient to induce potent effects in this model [47, 69]. In support, and along the dogma [21], an effective CT26-specific cytotoxic T cell response could be induced by this alternate route of infection or alternate bacterial agents (Figure 5).

How is this anti-tumor response effectively stimulated? It remains unclear whether it is locally induced in the tumor where bacteria are abundant, or in immune inductive sites by *Salmonella* that escape into the circulation within the initial hours of infection. Hence, it remains ambiguous whether adverse colonization provides the adjuvanticity needed to stimulate a preexisting anti-tumor response. We speculate that it could be a combination.

Tumor specificity for secondary tumors and metastases has been extensively demonstrated and described for bacteria, in particular *Salmonella* [31, 70–73]. It provides a solid argument for BMTT especially when combined with genetic cargo delivery [23, 74–79]. This ability has been tied to a systemic route of inoculation. Whether preserved with a local intra-tumor mode of infection remained uncertain. Indications supporting this possibility include an early TNF-α response, required to disrupt tumor vessels [20], and a window of opportunity where bacteria escape the primary tumor and circulate within the first hours of infection [47]. In our study, *Salmonella* was shown able to colonize secondary tumors subsequent to local inoculation of the primary tumor. As such, it exhibited equal potency by 48 hpi compared to systemic application. Thus, *Salmonella* exploits its tumor targeting ability in spite of local application.

Differences in tumor colonization between an i.t and an i.v. route of infection need to be considered. Bacterial application i.v. was shown to result in colonization of multiple CT26 tumors with a similar kinetic, whereas secondary tumors were invaded with a delay when i.t. infection was applied. However, a therapeutic difference of sooner or delayed tumor colonization remains elusive thus far. Preference could be dictated by requirements of i) the bacterial vector or ii) the genetic cargo. Minimizing transit time using local application may become desirable with highly virulent, toxic, inducible, or complement sensitive vectors. Conversely, the limitation of intra-tumoral application may be obvious with anatomically inaccessible tumors. In such cases, systemic infection may provide the only feasible solution although ultra sound guided injections might represent a possibility in some cases. Importantly however, the same plateau of tumor colonization, likely dictated by turnover and nutritional limitations [80], was achieved via either route of infection of both primary and secondary tumors (Figure 6). Surely, this would allow complete exploitation of an intrinsic tumor targeting ability of *Salmonella* for vehicular purposes. *Salmonella* provided superior adjuvanticity after adoptive transfer of tumor-specific syngeneic T cells [81].

The application of i.t. injections in an intradermal syngeneic mouse model for tumor therapy may question the usability of such a technique in more clinical relevant models or in the clinics. However, recent examples show that human patient-derived xenografts in immunodeficient mice are also sensitive to i.t. injections as previously demonstrated for i.v. infections [82]. Therefore, it would be of high interest to characterize the potential of i.t. injections for cancer therapy in such xenograft models that are already established [83–87]. In this context, the application of ultrasound and CT guided injections may facilitate targeting of deep tissue tumors, as shown recently and thus support a more general use of i.t. injection [88, 89].

In conclusion, intra-tumoral infection preserves the full therapeutic potential of *Salmonella* while providing substantial safety benefit and may be deployed to support a recombinant bacterial solution in the fight against cancer.

## EXPERIMENTAL PROCEDURES

### Ethics statement

All animal experiments were performed according to guidelines of the German Law for Animal Protection and with permission of the local ethics committee and the local authority LAVES (Niedersächsisches Landesamt für Verbraucherschutz und Lebensmittelsicherheit) under permission number 33.9-42502-04-12/0713.

### Strain development

Bacterial strains and plasmids are shown in Supplementary Table 1. Bacteria were grown in LB medium at 37°C. P22 bacteriophage transduction was used for targeted gene deletions [90]. Deletion of ydiV or ssrA was introduced to SF102 yielding SF199 or SF201, respectively. Refer to Supplementary Table 1 for genotypes. To obtain SF200, Δ*fliF::Frt-Kanamycin-Frt (FKF)* was transduced into SF199. For live imaging purposes, plasmid pHL304 encoding the *luxCDABE* operon conferring constitutive Lux-expression was transformed into bacteria via electroporation.

### Preparation of inoculum

*Salmonella* and *E. coli* strains were grown overnight and sub-cultured to mid-log phase in LB media at 37°C. Symbioflor-2 was adjusted as described previously [47]. In general, the bacteria were washed twice and adjusted to the desired OD_600_ in pyrogen free PBS.

### Murine tumor model

Six to seven week old BALB/c mice (Janvier) were intradermally inoculated with 5×10^5^ syngeneic CT26 tumor cells (colorectal cancer, ATCC CRL-2638) or 5×10^5^ F1.A11 tumor cells (fibrosarcoma) in the right flank. Tumor development was monitored using caliper measurements. Upon reaching a tumor volume of approx. 150 mm^3^ after 10 days, the mice were injected intravenously into the tail vein with 5×10^6^ *Salmonella* or *E. coli*, unless otherwise specified.

### Therapeutic benefit and bacterial burden

Tumor development was monitored using caliper measurements for as long as tumors persisted or until confronted with a humane endpoint in terms of exceedingly large tumor size or morbidity. Body weight was monitored and used as general health indicator. A loss of body weight below 80% of the original body weight was incentive to euthanize a mouse. To determine the bacterial burden, blood, spleen, liver and tumors were harvested at 36 hours post infection and treated as described previously [13]. CFUs were counted and the bacterial burden was calculated as total CFU per gram tissue.

### TNF-α measurement in serum

Blood samples were collected 1.5 and 3 h post infection. The TNF-α ELISA MaxTM Standard Kit (Biolegend) was used to determine the TNF-α level in serum. All steps were done according to the manufacturer's manual. Three different biological replicates were analyzed and a PBS treated group served as negative control.

### Histology

Upon isolation, specimens were fixed with 4% (v/v) formalin for 24 – 48 h, embedded in paraffin. Approximately 3 μm thick sections were stained with hematoxylin/ eosin according to standard procedures. Immuno-histo-chemical staining was performed using the following antibodies: rabbit-anti-pimonidazole (HP3-100 kit, Hydroxyprobe inc.), rabbit-anti-MPO (Medac/ Thermo Scientific), rabbit-anti-salmonella (US Biological) and DAB (3, 3-Diaminobenzidine Zytomed Systems DAB530) as chromogen. Sections were analyzed by light microscopy blinded to the experimental groups.

### Non-invasive *in vivo* imaging

For real-time observation of bacterial distribution, infections were carried out with luminescent bacteria, constructed as described under “strain development”. Prior to Image acquisition via the IVIS200 System (Calipers), mice were anaesthetized with 2% Isoflurane using the XGI-8 gas system (Calipers). Image analysis was performed using the Living image 3.0 software (Xenogen).

### Statistics

Significance between two groups was determined using the nonparametric Mann-Whitney test, while one-way analysis of variance (ANOVA) with Bonferroni posttest was used to compare two or more groups. Significance levels of p < 0.05, p < 0.01, or p < 0.001 were denoted with asterisks: *, **, and ***, respectively.

## SUPPLEMENTARY MATERIALS FIGURES AND TABLE


