# Supplementary material for: Local application of bacteria improves safety of Salmonella-mediated tumor therapy and retains advantages of systemic infection
Source: Oncotarget. 2017 Jun 7;8(30):49988–50001. doi: 10.18632/oncotarget.18392 (PMC5564822; doi:10.18632/oncotarget.18392)
Supplement: Supplementary file 1 [file oncotarget-08-49988-s001.pdf]

Local application of bacteria improves safety of *Salmonella* -mediated tumor therapy and retains advantages of systemic infection

SUPPLEMENTARY MATERIALS

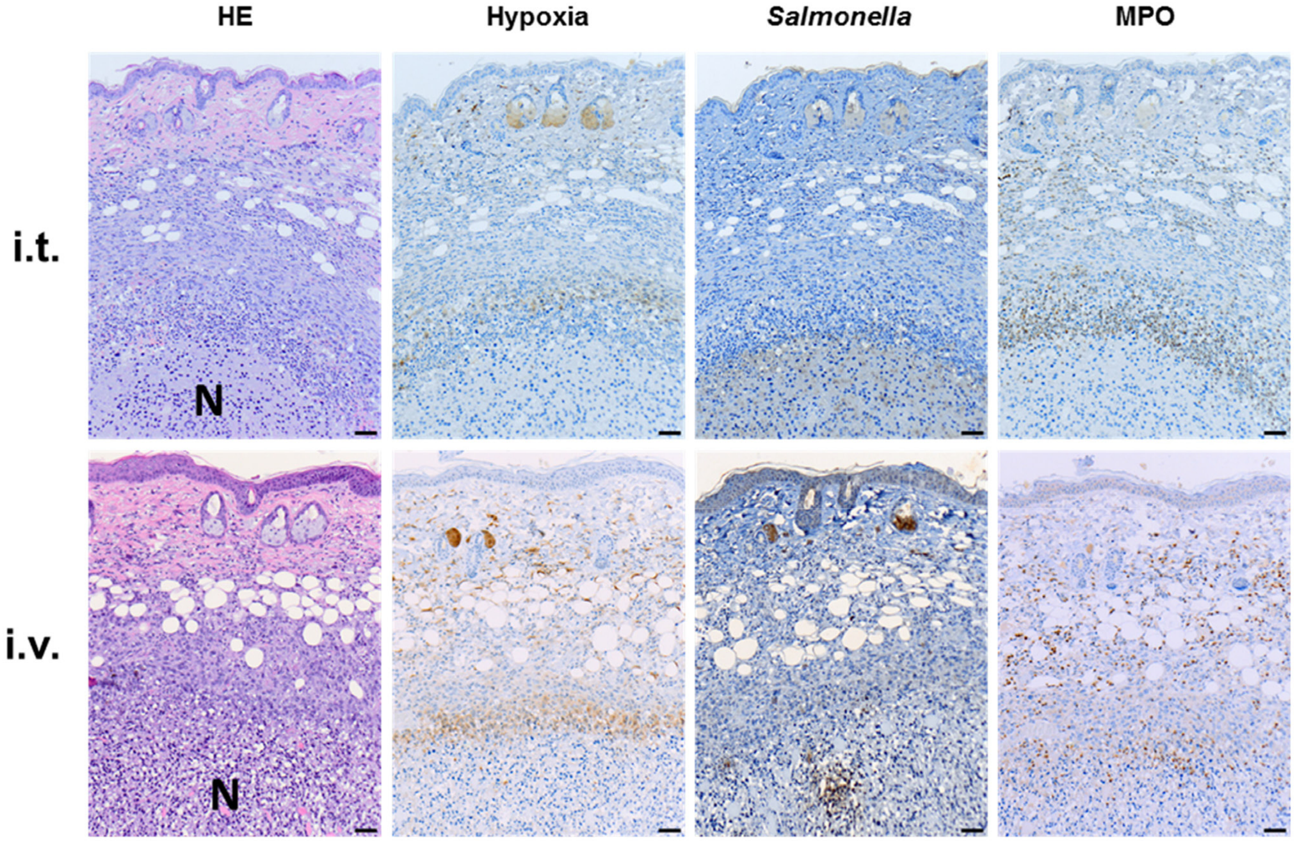

Supplementary Figure 1: Intra-tumoral inoculation of *Salmonella* causes manifestations in the CT26 tumor similar to systemic infection at 24 hpi. CT26 tumor-bearing mice were infected with 5x10^6 SF200 (*ΔlpxR9 ΔpagL7 ΔpagP8 ΔaroA ΔydiV ΔfliF*) via i.t. and i.v. routes of inoculation (top and bottom row, resp.). 24 hpi, tumors were isolated, embedded in paraffin and prepared for immune histochemical staining. Similar histological profiles between i.t. and i.v. infections: similar degree of necrosis formation and hypoxia, dispersion of salmonellae in and beyond necrotic center, and presence of neutrophils in immediate proximity to the salmonellae. Images displayed are representative of four replicates. "N" denotes area of necrosis. Hypoxia was stained with antibodies against metabolites of pimonidazole-HCl administered i.v. 30 mins prior to isolation. Myeloperoxidase (MPO) denotes presence of neutrophilic granulocytes, and Salmonella was stained using a specific antibody. Differential staining was performed on consecutive sections. Scale bar corresponds to 100 µm. Images representative of at least 3 replicates are displayed.

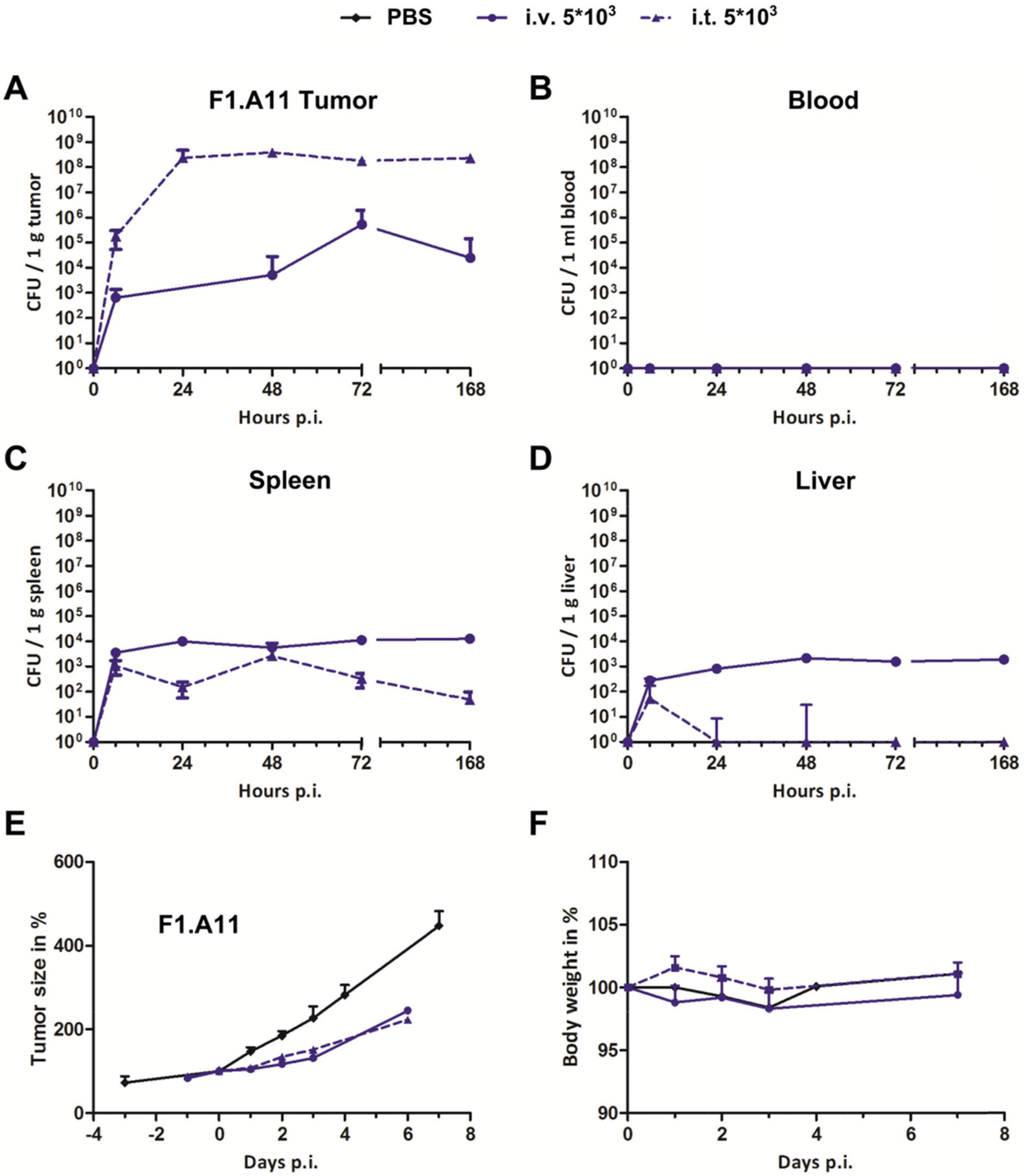

Supplementary Figure 2: Low dose Intra-tumoral infection with *Salmonella* limits adverse colonization without compromising therapeutic effects. CT26- tumor bearing mice were infected i.v. and i.t. with a dose of 5x10³ *Salmonella* variant SF201 (*ΔlpxR9 ΔpagL7 ΔpagP8 ΔaroA ΔssrA::Km*). Colonization of tumors **(A)**, blood **(B)**, spleen **(C)** and liver **(D)**. Bacterial burden was determined by plating serial dilutions of tissue homogenates. CFU were analyzed 36 hpi. **(E)** Tumor volumes were calculated on the basis of caliper measurements following infection with SF201. PBS served as negative control. **(F)** Body weight measurement served as indicator for the general health status. I.t. infection resulted in a significantly reduced bacterial burden and body weight decrease. PBS served as negative control. Displayed are medians with range. Results are representative of two independent experiments with five replicates per group.

Supplementary Table 1: Bacterial strains and plasmids used in this study

Strain	Description	Source	Ref.
***Salmonella* Typhimurium strains**			
**SF102**	*ΔlpxR9 ΔpagL7 ΔpagP8 ΔaroA*	-	[1]
**SF199**	SF102 + *ΔydiV*	This study	-
**SF200**	SF102 + *ΔydiV ΔfliF:Frt-Kanamycin-Frt (FKF)*	This study	-
**SF201**	SF102 + *ΔssrA::km*	This study	-
**SF202**	SF102 + *ΔydiV ΔfliF:FKF pHL304*	This study	-
***E. coli* strains**			
**EcN**	*Escherichia coli* Nissle 1917 (Mutaflor)	Lab stock	-
**Symbioflor-2**	*Escherichia coli* Symbioflor-2	SymbioPharm	-
	(G1/2, G3/10, G4/9, G5, G6/7 and G8, pooled 1:1)		
**Plamids**			
**pHL304**	*luxCDABE, Amp+, Plac*	Lab stock	[2]

1. Felgner S, Frahm M, Kocijancic D, Rohde M, Eckweiler D, Bielecka A, Bueno E, Cava F, Abraham WR, Curtiss R, Häussler S, Erhardt M, Weiss S. aroA-deficient *Salmonella enterica* serovar typhimurium is more than a metabolically attenuated mutant. MBio. 2016; 7:e01220-16.
2. Loessner H, Leschner S, Endmann A, Westphal K, Wolf K, Kochruebe K, Miloud T, Altenbuchner J, Weiss S. Drug-inducible remote control of gene expression by probiotic *Escherichia coli* Nissle 1917 in intestine, tumor and gall bladder of mice. Microbes Infect. 2009; 11:1097-105.
